# Supplementary material for: A pilot pragmatic randomized controlled trial of a 12-month Healthy Lifestyles Program: A collaborative care model for chronic conditions addressing behavioural change
Source: PLoS One. 2025 May 14;20(5):e0322118. doi: 10.1371/journal.pone.0322118 (PMC12077671; doi:10.1371/journal.pone.0322118)
Supplement: S1 Table — (PDF) [file pone.0322118.s001.pdf]

S1 Table. Descriptive and outcome measures.

Measure	Details	Data collection method	Timepoints
**FEASIBILITY (**Primary objective)			
Recruitment	Number of participants reaching out to program and number attending information sessions and eligible to participate in study.	Directly measured from administrative data	Baseline
Participation rate	Percent of participants that started the program after allocation to either the intervention or comparator group.	Directly measured from administrative data	Baseline
Retention rate	Percent of participants who completed the program 12-months after allocation.	Directly measured from administrative data	12-months
Attendance rate	Participation in individual sessions with interventionists and weekly psycho-educational group sessions (intervention group only).	Directly measured from administrative data	12-months
Recruitment methods	Qualitative data collected from participant feedback surveys regarding recruitment sources and reasons for enrolment.	Self-reported	Baseline
Missing data	Percent of incomplete data collected.	Directly measured from administrative data	12-months
**IMPLEMENTATION (using RE-AIM)** (Secondary objective)			
**Reach**			
Baseline Demographics	Age, gender, marital status, education level, employment status, household income, number and types of chronic conditions (multimorbidity defined as 2+ chronic conditions)	Self-reported	Baseline
Modifiable risk behaviours	Tobacco use, recreational drug use, alcohol consumption, physical activity	Self-reported	Baseline
**Effectiveness**			
Goal Attainment Scores	Personalized goals where participants indicate level of attainment on a 7-point Likert scale, where 1 represents worst-case scenario and 7 represents best-case scenario.	Self-reported	Baseline, 3,6,9 and 12-months
Anthropometric Measures	Body Mass Index (BMI), systolic BP, diastolic BP, waist circumference, hip circumference, and waist-hip ratio.	Directly measured	Baseline, 3,6,9 and 12-months
Mental health outcomes			
• Depression (PHQ9)	Patient Health Questionnaire 9-item scale (PHQ9) assesses depression severity as a continuous measure from 0 to 27. Threshold scores of 5, 10, 15, and 20 correspond to mild, moderate, moderately severe, and severe depression, respectively.	Self-reported	Baseline, 3,6,9 and 12-months
• Anxiety (GAD-7)	General Anxiety Disorder 7-item scale (GAD-7) assesses anxiety severity as a continuous measure ranging from 0 to 21. Threshold scores of 5, 10, and 15 correspond to mild, moderate and severe anxiety, respectively.	Self-reported	Baseline, 3,6,9 and 12-months
• Insomnia (ISI)	Insomnia Severity Index (ISI) is a 7-item questionnaire assessing insomnia severity on a scale from 0 to 28. Threshold scores of 8, 15, and 22 correspond to	Self-reported	Baseline, 3,6,9 and 12-months

	subthreshold, moderately severe and severe insomnia, respectively.		
• Stress (LCIS)	The Life Change Index (LCIS), is a 43-item tool used to measure stress levels, with scores ranging from 0 to 430 points. A score below 150 indicates low stress, while scores between 150 and 299 suggest moderate stress. Scores exceeding 300 are classified as high stress, indicating a significant risk of stress-related health issues.	Self-reported	Baseline, 3,6,9 and 12-months
• Stress (PSS-10)	The Perceived Stress Scale (PSS-10) is a 10-item questionnaire with a total score ranging from 0 to 40. Scores below 13 indicate low stress, 14–26 indicate moderate stress, and scores above 27 indicate high stress.	Self-reported	Baseline, 3,6,9 and 12-months
• Stress (PSS-4)	The Perceived Stress Scale (PSS-4) is a 4-item questionnaire with a total score ranging from 0 to 16. Scores below 5 indicate low stress, 5–9 indicate moderate stress, and scores above 9 indicate high stress.	Self-reported	Baseline, 3,6,9 and 12-months
• Loneliness	The DeJong Gierveld Loneliness Scale is a 6-item questionnaire that was modified from a 6-point to 12-point scale. Higher scores indicate greater loneliness with subscales for emotional loneliness and social loneliness.	Self-reported	Baseline, 3,6,9 and 12-months
• Health Related Quality of Life - RAND SF-36	The RAND SF-36 is a 36-item questionnaire assessing overall health status, with scores from 0 to 100, where 100 indicates the best health and 0 the worst. It evaluates eight health domains: physical functioning, role limitations (physical and emotional), energy/fatigue, emotional well-being, social functioning, pain, and general health. Additionally, it provides two composite scores for physical and mental health, making it a valuable tool for tracking health outcomes and evaluating interventions.	Self-reported	Baseline, 3,6,9 and 12-months
• Health Related Quality of Life-HUI2	The Health Utility Index Mark 2 (HUI2) is characterized by six attributes: sensation (vision, hearing, and speech), mobility, emotion, cognition, self-care, and pain. The overall utility scores for HUI2 ranges from -0.03 to 1.0, with -0.03 representing a health state worse than death, 0.0 representing death, and 1.0 representing perfect health.	Self-reported	Baseline, 3,6,9 and 12-months
• Health Related Quality of Life-HUI3	The Health Utility Index Mark 3 (HUI3) is characterized by eight attributes: vision, hearing, speech, ambulation, dexterity, emotion, cognition, and pain. The overall utility scores for HUI3 ranges from -0.36 to 1.0, where -.36 represents a health state worse than death, 0.0 representing death, and 1.0 representing perfect health.	Self-reported	Baseline, 3,6,9 and 12-months
Participant Satisfaction	Participants evaluated their perceived satisfaction, enjoyability and usefulness of the program using a 5-point Likert scale, where 1 represented the lowest rating and 5 the highest.	Self-reported	3,6,9 and 12-months
**Adoption**			
Qualitative Data	Semi-structured interviews with interventionists and participant's healthcare providers.	Researcher-administered	6 and 12 months
**Implementation**			
Qualitative Data	Semi structured interviews with participants, interventionists, and participant family members.	Researcher-administered	Interventionists – 6 and 12 months

			Family members – 9 months<br>Participants – 12 months
