# Supplementary material for: A pilot pragmatic randomized controlled trial of a 12-month Healthy Lifestyles Program: A collaborative care model for chronic conditions addressing behavioural change
Source: PLoS One. 2025 May 14;20(5):e0322118. doi: 10.1371/journal.pone.0322118 (PMC12077671; doi:10.1371/journal.pone.0322118)
Supplement: S2 Table — (PDF) [file pone.0322118.s002.pdf]

S2 Table. Within-group analysis of outcomes for participants in the intervention group (n=9) and comparator group (n=7) using GEE and adjusting for gender and age.

Outcome	Intervention Group (n=9)				Comparator Group (n=7)				MCID
	Baseline	12-months	Change		Baseline	12-months	Change		
	Mean (SD)	Mean (SD)	β	95%CI	Mean (SD)	Mean (SD)	β	95%CI	
Mental Health									
Insomnia Severity Index (ISI)	9.56 (5.00)	5.33 (4.92)	-1.19	-2.04 to -0.33	9.71 (4.39)	8.00 (3.51)	-0.45	-0.81 to -0.09	6
Patient Health Questionnaire 9	7.56 (5.27)	3.00 (1.73)	-1.01	-1.76 to -0.27	6.7 (4.72)	5.43 (2.99)	-0.27	-0.86 to 0.33	5
Perceived Stress Index – 4 Item (PSS4)	5.44 (3.00)	2.56 (1.13)	-0.670	-1.12 to -0.22	5.43 (3.15)	4.29 (1.89)	-0.25	-0.69 to 0.19	ND
Perceived Stress Index – 10 Item (PSS10)	14.89 (5.82)	8.67 (3.74)	-1.44	-2.31 to -0.57	14.57 (5.68)	11.14 (4.74)	-1.00	-1.75 to -0.25	2 to 4
Life Change Index (LCI)	290 (245)	217 (131)	-16.87	-67.88 to 32.15	501 (506)	446 (331)	-11.12	-32.45 to 10.22	ND
DeJong Gierveld Total Score (loneliness)	3.22 (2.82)	0.56 (0.88)	-0.48	-0.76 to -0.21	3.00 (3.92)	2.43 (2.99)	-0.14	-0.40 to 0.12	ND
Goals									
Number of Active Goals	2.67 (0.71)	3.67 (1.12)	0.20	0.07 to 0.22	1.71 (0.49)	2.57 (0.79)	0.21	0.10 to 0.33	ND
Goal Attainment Score	1.81 (0.60)	5.40 (0.96)	1.06	0.89 to 1.23	2.07 (1.10)	4.21 (0.99)	0.59	0.35 to 0.84	1
Rand SF-36 (health related quality of life)									
Physical Functioning	48.33 (31.22)	58.33 (34.00)	2.48	-2.31 to 7.27	67.14 (28.12)	75.00 (19.79)	1.96	-0.73 to 4.65	10
Role Limitation due to Physical Health	36.11 (48.59)	52.77 (40.40)	5.02	-1.67 to 11.71	46.43 (44.32)	60.71 (49.70)	3.05	-5.78 to 11.88	10
Role Limitation to due Emotional Well-Being	48.15 (44.44)	55.56 (37.27)	1.85	-0.91 to 4.62	61.90 (40.50)	57.14 (41.79)	-0.02	5.78 to 5.74	10
Energy/ Fatigue	38.89 (25.59)	52.22 (10.34)	3.08	-0.66 to 6.81	45.71 (17.66)	57.86 (18.00)	3.10	-0.93 to 7.12	10
Emotional Well-Being	72.44 (14.76)	79.11 (7.15)	1.13	-0.16 to 2.41	71.43 (20.97)	71.43 (13.15)	0.95	-1.91 to 3.81	10
Social Functioning	58.33 (33.66)	72.22 (19.54)	3.24	-0.02 to 5.50	64.29 (32.62)	71.43 (21.30)	2.36	-1.69 to 6.41	10
Pain	51.67 (27.39)	68.33 (27.78)	4.30	1.52 to 7.09	49.64 (31.83)	53.93 (24.50)	0.97	-2.42 to 4.35	10
General Health	35.00 (14.58)	57.22 (25.01)	5.57	2.02 to 9.12	57.86 (25.63)	65.71 (22.81)	1.95	1.13 to 2.76	10
Physical Composite Score	34.11 (11.81)	41.72 (14.09)	1.95	0.06 to 3.85	40.31 (11.32)	45.27 (6.14)	1.19	-0.22 to 2.61)	2 to 4
Mental Composite Score	42.73 (8.12)	46.63 (7.52)	0.29	-1.60 to 2.18	44.33 (13.58)	45.04 (11.72)	0.43	(-1.31 to 2.17	2 to 4
Health Utility Index (health related quality of life)									
HUI3 Composite Score	0.44 (0.33)	0.59 (0.28)	0.03	-0.01 to 0.08	0.54 (0.28)	0.62 (0.22)	0.02	-0.02 to 0.05	0.03
HUI2 Composite Score	0.61 (0.29)	0.69 (0.18)	0.02	-0.01 to 0.05	0.67 (0.30)	0.70 (0.24)	0.01	-0.02 to 0.03	0.03
HUI General Health	3.78 (0.97)	3.33 (1.22)	-0.15	-0.39 to 0.09	2.86 (0.69)	2.57 (0.79)	-0.07	-0.20 to 0.06	0.05
Anthropometric Measures									
Systolic Blood Pressure (mmHG)	126.57 (9.83)	131.14 (19.12)	-19.88	-46.97 to 7.22	123.86 (20.47)	120.29 (18.63)	-1.24	-2.97 to 0.48	2
Diastolic Blood Pressure (mmHG)	83.29 (9.55)	79.00 (8.45)	-24.55	-54.10 to 5.00	79.86 (7.65)	76.57 (10.11)	-0.90	-2.70 to 0.89	2
Body Mass Index (BMI) (kg/m^2)	42.33 (8.52)	42.55 (7.89)	0.05	-0.20 to 0.30	30.04 (5.25)	30.17 (5.24)	0.03	-0.30 to 0.37	5 to 10%

Hip circumference (cm)	134.20 (16.89)	135. 62 (17.48)	0.36	-0.49 to 1.20	114.31 (7.49)	112.57 (10.31)	-0.37	-1.32 to 0.58	ND
Waist circumference (cm)	127.91 (21.24)	125.48 (21.27)	-0.63	-2.03 to 0.77	104.86 (7.58)	105.71 (7.78)	0.23	-0.93 to 1.39	ND
Waist-hip ratio	0.95 (0.05)	0.92 (0.07)	-0.01	-0.02 to -0.01	0.92 (0.05)	0.94 (0.04)	0.01	0.001 to 0.01	ND

CI- Confidence Interval; MCID – Minimal clinically important difference; ND – Not determined; SD- Standard Deviation
