# Supplementary material for: A pilot pragmatic randomized controlled trial of a 12-month Healthy Lifestyles Program: A collaborative care model for chronic conditions addressing behavioural change
Source: PLoS One. 2025 May 14;20(5):e0322118. doi: 10.1371/journal.pone.0322118 (PMC12077671; doi:10.1371/journal.pone.0322118)
Supplement: S3 Table — (PDF) [file pone.0322118.s003.pdf]

S3 Table. Between group analysis for patient-centred outcomes (N=16) using GEE analysis and adjusting for age and gender.

OUTCOMES (N=16)<br>Comparator group used as reference	Effect (β)	95% CI	
		Lower	Upper
**Mental Health**			
Insomnia Severity Index	-2.48	-5.75	0.79
Patient Health Questionnaire 9	-4.08	-7.23	-0.94
Perceived Stress Index – 4 Item scale	-1.54	-4.16	1.09
Perceived Stress Index – 10 Item scale	-2.40	-7.33	2.53
Life Change Index	-28.79	-208.20	150.61
DeJong Gierveld Score	-2.23	-3.88	-0.58
**Goals**			
Number of Active Goals	0.12	-0.40	0.64
Goal Attainment Score	1.36	0.51	2.20
**Rand SF-36**			
Physical Functioning	9.41	-6.42	25.24
Role Limitation due to Physical Health	3.29	-47.24	53.83
Role Limitation to due Emotional Well-Being	11.67	-25.92	49.26
Energy/ Fatigue	0.95	-23.82	25.72
Emotional Well-Being	6.90	-3.13	16.92
Social Functioning	7.28	-10.64	25.19
Pain	14.42	-3.32	32.15
General Health	16.92	3.38	39.47
Physical Composite Score	4.17	-4.68	13.02
Mental Composite Score	3.43	-5.29	12.14
**Health Utility Index (HUI)**			
HUI3 Composite Score	0.14	-0.04	0.31
HUI2 Composite Score	0.07	-0.06	0.20
HUI General Health	-0.29	-1.33	0.75
**Anthropometric Measures**			
Systolic Blood Pressure (mmHG)	-8.57	-18.85	1.53
Diastolic Blood Pressure (mmHG)	-0.09	-8.21	8.03
Body Mass Index (kg/m^2)	0.06	-1.97	0.76
Hip circumference (cm)	2.02	-1.63	5.67
Waist circumference (cm)	-3.77	-10.29	2.73
Waist-hip ratio	-0.05	-0.08	-0.16

CI- Confidence Interval
