# Supplementary material for: A pilot pragmatic randomized controlled trial of a 12-month Healthy Lifestyles Program: A collaborative care model for chronic conditions addressing behavioural change
Source: PLoS One. 2025 May 14;20(5):e0322118. doi: 10.1371/journal.pone.0322118 (PMC12077671; doi:10.1371/journal.pone.0322118)
Supplement: S4 Table — Analysis using GEE and adjusting for age and gender. (PDF) [file pone.0322118.s004.pdf]

S4 Table. Associations between attendance and outcomes for the intervention group participants (n=9). Analysis using GEE and adjusting for age and gender.

OUTCOMES (n=9)	Effect (β)	95% CI	
		Lower	Upper
**Mental Health**			
Insomnia Severity Index	-0.166	-0.227	-0.055
Patient Health Questionnaire 9	-0.135	-0.228	-0.42
Perceived Stress Index – 4 Item scale	-0.084	-0.146	-0.022
Perceived Stress Index – 10 Item scale	-0.187	-0.312	-0.062
Life Change Index	-2.974	-11.673	5.726
DeJong Gierveld Score	-0.075	-0.111	-0.038
**Goals**			
Number of Active Goals	0.035	0.013	0.057
Goal Attainment Score	0.117	0.060	0.174
**Rand SF-36**			
Physical Functioning	0.255	-0.227	0.737
Role Limitation due to Physical Health	0.322	-0.555	1.199
Role Limitation to due Emotional Well-Being	0.077	-0.393	0.548
Energy/ Fatigue	0.454	0.042	0.865
Emotional Well-Being	0.226	-0.040	0.492
Social Functioning	0.510	0.052	0.968
Pain	0.320	-0.057	0.697
General Health	0.867	0.411	1.341
Physical Composite Score	0.201	-0.004	0.406
Mental Composite Score	0.180	0.098	0.261
**Health Utility Index**			
HUI3 Composite Score	0.004	-0.002	0.010
HUI2 Composite Score	0.003	-0.001	0.007
HUI General Health	-0.020	-0.047	0.008
**Anthropometric**			
Systolic Blood Pressure (mmHG)	0.158	-0.154	0.470
Diastolic Blood Pressure (mmHG)	-0.080	-0.258	0.098
Body Mass Index (kg/m^2)	-0.012	-0.048	0.024
Hip circumference (cm)	-0.138	-0.351	0.075
Waist circumference (cm)	0.012	-0.098	0.123
Waist-hip ratio	-0.001	-0.002	0.000
