# Supplementary material for: A pilot pragmatic randomized controlled trial of a 12-month Healthy Lifestyles Program: A collaborative care model for chronic conditions addressing behavioural change
Source: PLoS One. 2025 May 14;20(5):e0322118. doi: 10.1371/journal.pone.0322118 (PMC12077671; doi:10.1371/journal.pone.0322118)
Supplement: Attachment — Submitted filename: Peer Review of HLP.docx [file pone.0322118.s005.docx]

Peer review of *“A Pilot Pragmatic Randomized Controlled Trial of a 12-month Healthy Lifestyles Program: A Collaborative Care Model for Chronic Conditions Addressing Behavioural Change*”

PLOS ONE

Thank you for the opportunity to review this manuscript, which describes a pilot feasibility study of the Healthy Lifestyles Program (HLP), a 12-month behavioural intervention designed to address lifestyle-related chronic diseases. This study makes a meaningful contribution by addressing the critical challenge of translating evidence-based lifestyle interventions into real-world settings. The manuscript is commendable for its use of an implementation evaluation framework (RE-AIM), inclusion of an active control group, and integration of mixed methods, which enhance the study's rigor and relevance. However, greater clarity, consistency and transparency in reporting the qualitative evaluation would significantly enhance the manuscript.

Specific Areas for Improvement:

**Abstract**

- The abstract refers to "content analysis" (line 61) for qualitative data, while the main manuscript describes the use of "thematic analysis". This inconsistency should be resolved to ensure transparency in methodology.

**Introduction**

- Lines 108–109 mention that the programme was informed by CBT principles and health behaviour theories. Expanding on how these principles guided programme development would enhance reproducibility and inform the reader's understanding of the intervention, though this is not essential.
- The manuscript could benefit from a visual diagram or table to clarify the differences between the control and intervention conditions, as there is some overlap.

**Methods**

- The inclusion of demographic data collection is a strength of this study, highlighting the authors' attention to participant characteristics. However, ethnicity is not reported. It is unclear whether ethnicity was recorded but not analysed, or omitted entirely. Given the known disparities in health outcomes and behavioural risk factors across racial and ethnic groups, this omission limits the assessment of the intervention’s feasibility and generalisability to diverse populations. I recommend the authors clarify:
  - Whether ethnicity data was collected but not reported, or not collected at all.
  - The rationale for any decisions regarding ethnicity data collection or reporting.
  - How future iterations of this work could address racial and ethnic disparities in both participation and outcomes to ensure equity in feasibility assessments and intervention design.
- The term "thematic analysis" appears here, contrasting with "content analysis" mentioned elsewhere. Provide a reference for the type of thematic analysis used in order to clarify the approach taken, ensuring consistency with its principles. For example, if Braun & Clarke’s thematic analysis was used, the consensus reaching approach described does not align with this methodology and divergences will need to be explained.
- The adjustment made to the scoring of the DeJong Gierveld Loneliness Scale to enhance sensitivity is an interesting and potentially valuable approach, but it may raise questions regarding its validity and comparability to other studies using the standard scoring system. Providing a brief justification for this adjustment and clarifying whether it was made a priori or post hoc would address these concerns.

**Results**

- A table of quotes is provided in Table 3, and the authors have performed a basic level of interpretation to connect the participant narratives to the broader RE-AIM framework. The themes identified are relevant, but some feel more descriptive than analytical (e.g., physical activity, MSK specialist). A brief explanation under each generated theme could provide greater confidence that a rigorous thematic analysis has been conducted, and help to link the participant quotes to the RE-AIM domains more explicitly.

**Discussion**

- It would strengthen the discussion if the authors considered potential reasons for the decrease in attendance over time. Did any of the qualitative findings indicate reasons for declining attendance or factors affecting 12-month retention rates?
- The authors attribute lower retention in the comparator group to insufficient interaction frequency (lines 485–486). While this is plausible, differences in job roles between intervention deliverers (a team of healthcare professionals) and comparator deliverers (a research assistant) should also be considered as a potential factor. Including this in the discussion would provide a more nuanced interpretation.
- If ethnicity data was indeed overlooked in this trial (see comment in methods section regarding this) then the limitations section should be expanded to include the absence of ethnicity data, which limits the evaluation of the intervention’s feasibility across diverse populations.
- In line 515, the authors propose changes to the intervention for scaling, such as online or self-paced sessions (line 515). While these ideas have merit, transitioning to self-guided formats could significantly alter the programme’s fundamental design and may not maintain the observed preliminary effectiveness. Instead, the authors should focus on exploring barriers to recruitment and retention while keeping the programme's core components intact.

**Summary:**

This manuscript provides valuable insights into the feasibility of implementing a collaborative care behavioural intervention for chronic conditions. The comprehensive use of the RE-AIM framework and the integration of mixed methods are particularly commendable. The study design is robust, and the results are presented clearly. However, improvements in the reporting and interpretation of the qualitative evaluation, alongside addressing some methodological and discussion points, would significantly enhance the manuscript’s overall quality.

I commend the authors for their innovative approach and for addressing an important gap in translating behavioural interventions into real-world settings. This work has great potential to inform future studies, and I hope my comments help strengthen the manuscript further.
